# Simple sequence repeats in *Haemophilus influenzae*

**DOI:** 10.1016/j.meegid.2008.11.006

**Published:** 2009-03

**Authors:** Peter M. Power, W.A. Sweetman, N.J. Gallacher, M.R. Woodhall, G.A. Kumar, E.R. Moxon, D.W. Hood

**Affiliations:** Molecular Infectious Diseases Group, Department of Paediatrics, Weatherall Institute of Molecular Medicine, University of Oxford, John Radcliffe Hospital, Oxford OX3 9DS, UK

**Keywords:** Phase variation, Genomes, *Haemophilus influenzae*

## Abstract

Simple sequence repeat (SSRs) of DNA are subject to high rates of mutation and are important mediators of adaptation in *Haemophilus influenzae*. Previous studies of the Rd KW20 genome identified the primacy of tetranucleotide SSRs in mediating phase variation (the rapid reversible switching of gene expression) of surface exposed structures such as lipopolysaccharide. The recent sequencing of the genomes of multiple strains of *H. influenzae* allowed the comparison of the SSRs (repeat units of one to nine nucleotides in length) in detail across four complete *H. influenzae* genomes and then comparison with a further 12 genomes when they became available. The SSR loci were broadly classified into three groups: (1) those that did not vary; (2) those for which some variation between strains was observed but this could not be linked to variation of gene expression; and (3) those that both varied and were located in regions consistent with mediating phase variable gene expression. Comparative analysis of 988 SSR associated loci confirmed that tetranucleotide repeats were the major mediators of phase variation and extended the repertoire of known tetranucleotide SSR loci by identifying ten previously uncharacterised tetranucleotide SSR loci with the potential to mediate phase variation which were unequally distributed across the *H. influenzae* pan-genome. Further, analysis of non-tetranucleotide SSR in the 16 strains revealed a number of mononucleotide, dinucleotide, pentanucleotide, heptanucleotide, and octanucleotide SSRs which were consistent with these tracts mediating phase variation. This study substantiates previous findings as to the important role that tetranucleotide SSRs play in *H. influenzae* biology. Two Brazilian isolates showed the most variation in their complement of SSRs suggesting the possibility of geographic and phenotypic influences on SSR distribution.

## 1 Introduction

*Haemophilus influenzae* (*Hi*), a common commensal bacterium of the upper respiratory tract of humans, is an important cause of diseases that include otitis media, pneumonia, meningitis, and septicaemia. The genome sequence of *Hi* strain Rd KW20, the first completed for a free-living organism, revealed a high prevalence of simple sequence repeats (SSRs) (Fleischmann et al., 1995; Hood et al., 1996b). SSRs are usually defined as direct, perfect DNA repeats consisting of repeat units (the smallest repeating DNA motif of the SSR) of between one and nine nucleotides in length. In many organisms, taking into account the nucleotide sequence composition of their respective genomes, SSRs are found less frequently than predicted (Mrázek et al., 2007). SSRs are hypermutable (e.g. tetranucleotide SSRs lose and gain units at a rate of 1 × 10^−4^ per generation (De Bolle et al., 2000) compared with a basal mutation rate of approximately 1 × 10^−9^) and, therefore, it has been suggested that their decreased prevalence reflects natural selection because the higher rates of mutation of these loci would be more often detrimental to fitness than beneficial. However, in some prokaryotes, predominantly host-adapted organisms, some SSRs are found in greater numbers than would be expected by chance (Mrázek et al., 2007). Analysis of SSRs in the *Hi* strain Rd KW20 genome revealed that long tracts of tetranucleotides were over-represented (Hood et al., 1996b). A striking feature of these tetranucleotide SSRs is their frequent association with genes whose functions are associated with microbial-host interactions relevant to commensal and virulence behaviour (Hood et al., 1996b).

SSRs can be located in promoter regions or within open reading frames and changes in their length can result in the random, high frequency, reversible loss, gain or modulation of gene expression (phase variation). Since these regions of localised hypermutation, often termed ‘contingency loci’, can each independently result in altered gene expression, a repertoire of phenotypic variants is generated (Moxon et al., 2006). Through selection of these variants, the adaptation of the bacterial population to changes in the host environment is facilitated. It has been suggested that this strategy has particular survival value when bacterial populations are subjected to periodic selection during transmission between genetically distinct hosts (Wolf et al., 2005).

The advent of the genomic sequencing of multiple strains of the same species has revealed that the genomic sequence of a particular strain may not reflect the diversity and variety of the entire species. The term ‘pan-genome’ has been used to describe the superset of genes of a species (Tettelin et al., 2005). The characterisation of a pan-genome describes the core (genes contained in all genomes of a species) and dispensable genes (those genes absent from one or more strains or unique to each strain) of a species. We suggest that the concept of a pan-genome should also include explicit recognition of differences in gene sequence, organisation and variation that may better describe the adaptive and evolutionary potential of the species (Caporale, 2006). In this study, we have sought to identify the potential repertoire of variation mediated by SSRs in the currently available *Hi* pan-genome.

Prior to this study, our understanding of SSRs in *Hi* has been predominantly based on analysis of the strain Rd KW20 genome sequence. Whilst selective studies of other *Hi* strains have provided some evidence to suggest variation in the number, location and nature of the SSRs compared to that seen in the Rd KW20 genome (Fox et al., 2005; van Belkum et al., 1997), the recent availability of a number of completely sequenced *Hi* genomes has provided us with the opportunity for a much more extensive analysis of SSRs in *Hi*.

We describe in detail 223 SSRs identified in the four complete genome sequences of strains RdKW20, 86-028NP, R2846 and R2866 plus 765 SSRs identified in the complete or partial genome sequences of a further 12 *Hi* strains. Previous reports of SSRs in *Hi* have been predominantly of tetranucleotide repeats. From these 16 genomes we describe 199 tetranucleotide SSRs in 28 different loci including 10 which have not previously been described. However, we have also identified a number of mononucleotide, dinucleotide, pentanucleotide, heptanucleotide, and octanucleotide SSRs with a putative role in phase variable gene regulation. A preponderance of the novel SSRs identified occur in only two strains, F3031 and F3034 of the *Hi* biogroup aegyptius, suggesting that the distribution of SSRs across the *Hi* pan-genome may be linked with geographic and phenotypic profiles.

## 2 Materials and methods

The four *Hi* genome sequences that were available at the commencement of this study formed the basis of the *Hi* four genome study. Details of these genomes are given in Table 1. A list of SSRs with repeat unit lengths of between one and nine nucleotides, and a number of repeat unit iterations above an empirically determined threshold value (see below) was compiled for each of these genomes using a PERL script that we have developed and have called *Hi*SSRfinder. The results from *Hi*SSRfinder were used to generate an annotated EMBL file for each of the genomes which then allowed manual analysis and curation of the SSRs identified in each genome using the Artemis and ACT genome viewing, annotation and comparison programs (Rutherford et al., 2000). Each SSR was manually evaluated with regards to its position relative to open reading frames (ORFs), whether an equivalent SSR was present or not in the other three strains and whether there was any variation in the SSR between strains (see Supplementary Table 1).

The threshold values, i.e. the minimum number of repeat units required to be present in an uninterrupted tandem arrangement within a genome in order for that sequence to be counted as an SSR and included in further analysis, was determined for each different length of repeat unit from a comparison of the number of SSRs of different lengths and the frequency of polymorphisms between the four genomes (see Section 3 and Table 2). The thresholds determined in this way for this study were as follows: 1 (repeat unit length), >8 (threshold value of repeat units); 2, >4; 3, >3; 4, >2, 5, >2; 6, >2; 7, >2; 8, >2 and 9, >2.

A database, named SSR_*Hi*_4G, was constructed to contain the nucleotide sequences of each of the tetranucleotide SSR, together with their 500 bp upstream and downstream flanking sequences, identified in the four genome study. This database was assembled using the formatdb program (SSR_Hi_4G is available at http://users.ox.ac.uk/∼oxmicro/ssrblast.html, formatdb program is available at http://www.ncbi.nlm.nih.gov/blast/download.shtml).

A second collection of *Hi* genomes (herein termed the further 12 genome study) was then examined using the information from the initial four genome survey to guide analysis. Details of these additional genomes are given in Table 1. The SSRs in these genomes were identified using the *Hi*SSRfinder script as described above. In order to determine which of the tetranucleotide SSRs identified in the 12 further genome sequences were equivalent to the tetranucleotide SSRs previously identified in the four genome study, each was compared to the SSR_*Hi*_4G database using the BLASTN program. Data and boxplot analysis of SSR data was performed using the R statistical package (http://www.r-project.org/) and Microsoft Excel. Transmembrane helicies were predicted using the TMHMM web server v2.0 (http://www.cbs.dtu.dk/services/http://www.cbs.dtu.dk/services/TMHMM; Moxon et al., 2006; Sonnhammer et al., 1998).

## 3 Results

### 3.1 Determination of threshold values used to identify SSRs in this study

Previous studies on *Hi* have described the SSRs present within the genome of strain Rd KW20 (Hood et al., 1996b). Our aim was to extend the analysis of SSRs by comprehensively investigating the repertoire present in the four complete *Hi* genome sequences that were available for different strains of *Hi* at the commencement of this study (Four Genome Analysis; see Table 1). In this study, SSRs are defined as tandem repeats of a repeat unit that consists of between one and nine nucleotides. In order to attain maximum sensitivity for the detection of SSRs the threshold values (see Section 2) were set as low as practically possible. Our rationale for adopting threshold values is described in Table 2, which shows the number of SSRs identified in the genome of strain Rd KW20 at the threshold values adopted and also the number of SSRs that would have been included in the subsequent analysis if the threshold value had been set one unit higher or lower for each repeat unit length. It can be seen from Table 2 that, for all but the tetranucleotide SSRs, increasing the threshold value by one substantially decreased the number of SSRs detected and resulted in a total of only 19 SSRs being identified in the Rd KW20 genome. In contrast, decreasing the threshold by one resulted in a large number of SSRs being identified which would be impractical for manual analysis (3924 in strain Rd KW20). At the adopted threshold values, 60 SSRs were identified in strain Rd KW20. The thresholds used in this study for all repeat unit lengths included at least all of the statistically unexpected SSRs determined by hidden Markov model analysis of the *Hi* genomes (see Table 2; Paul Swift, Oxford, personal communication). This further substantiates the threshold values chosen as being permissive for having a high degree of sensitivity in identifying SSRs with potential roles in mediating phase variation. Additionally, if SSRs were found to be above threshold in at least one genome the corresponding regions in the other genomes were also characterised.

A total of 223 SSRs were identified in the four genome sequences when the threshold values described in Table 2 were applied and these 223 SSRs are summarised in Table 3. Comparison of the SSRs across the four *Hi* genomes for each of the repeat unit lengths, reveals that their numbers are not substantially different between the strains. Also, the total number of repeats found within any one strain is not substantially different from the others (total number of SSRs ranged between 53 and 60), despite the differences in the origin, associated disease and date of isolation of the four strains (see Table 1).

SSRs have previously been associated with hypermutation, as loss or gain of repeat units occur at high frequency due to replication slippage (Moxon et al., 2006). Loss or gain of repeat units from an SSR located within an ORF may result in a frameshift mutation if the length of the repeat unit is not a multiple of three. The position of each of the 223 SSRs identified in this study was manually curated and the proportion of SSRs that were located within ORFs was recorded (see Table 3). *Hi* has a coding density of approximately 88% of the genome sequence and, of the repeats examined, only the trinucleotide (83%), tetranucleotide (100%) and hexanucleotide (87%) SSRs occur within ORFs at approximately this frequency, whilst SSRs with repeat unit lengths of one, two, five and seven nucleotides were all found to be located within ORFs with a frequency of less than 88%. SSRs with longer repeat unit lengths were not included in this analysis due to their low frequency in the genomes.

This suggests that the selective pressure against trinucleotide and hexanucleotide SSRs occurring within an ORF may not be as high as that against SSRs of other repeat unit lengths whose expansion or contraction would result in inactivation of an ORF by frameshift mutation. It is noteworthy that tetranucleotide SSRs are found exclusively within ORFs, consistent with the known importance of this class of repeat in mediating phase variable expression at contingency loci in *Hi*.

### 3.2 Identification of repeat unit lengths likely to be associated with phase variable gene expression

Manual curation of each of the 223 SSRs allowed us to assess the likelihood of each SSR playing a role in modulating gene expression. Comparison of equivalent SSR loci (those located in the same relative genomic location) allowed the classification of each SSR into one of three categories: (1) SSRs that did not vary in length, sequence or position between the four genomes (invariable), (2) SSRs for which some variation between strains was observed but the variation was not considered likely to result in variation of gene expression (variable) and (3) SSRs that both varied in length and were located in regions consistent with mediating phase variation (potentially phase variable; see Table 4). Careful manual examination of each of the repeat associated loci was necessary to classify the SSRs into the above categories. Factors such as the location of the SSR within a gene, length of the SSR and replacement of a whole or partial tract of an SSR by another sequence contributed to the assessment of whether or not any observed variation in the SSR was likely to mediate phase variation. SSRs located outside ORFs were generally more difficult to assess as to their likely involvement in phase variable modulation of gene expression. SSRs have previously been shown to be mediators of phase variation through modulation of promoter activity and gene transcription (Dawid et al., 1999; Martin et al., 2005; van Ham et al., 1993), but promoter regions of individual genes often cannot be accurately defined. Thus, the influence of variation in SSRs located in non-coding regions on expression of adjacent genes is difficult to predict. The full assessment of the 223 SSRs identified within the four genomes can be found in Supplementary Table 1; a summary of the data is provided in Table 4.

The manual classification of the SSRs into the three categories indicated that despite the considerable variation seen between strains for many of the SSRs (especially the mononucleotide SSRs), the tetranucleotide, pentanucleotide and heptanucleotide repeat tracts were the only types of SSR considered to have a potential role in mediating phase variable gene expression in these four strains of *Hi* (Table 4). The potentially phase variable ORFs associated with each of these types of repeat are detailed below.

### 3.3 Tetranucleotide SSRs identified in the *Hi* four genome study

A previous analysis of the Rd KW20 genome sequence identified the primacy of tetranucleotide SSR in mediating phase variation in *Hi* (Hood et al., 1996b). This study extends that work by comparing the tetranucleotide SSRs across four *Hi* genomes. We identified 18 different tetranucleotide SSR loci that are distributed fairly uniformly between genomes with each genome containing from 12 to 14 tetranucleotide SSR loci (Table 5). Eight of the tetranucleotide SSR loci were found in all four of the strains, two of the loci were found in three of the strains, three of the loci in two of the strains and five of the loci were unique to one strain (two unique loci in each of the strains Rd KW20 and 86-028NP and one unique locus in R2846).

Two of the tetranucleotide SSR loci that we have identified in the four genome analysis have not previously been described as potential mediators of phase variation in *Hi*. The first of these novel loci contains 14 tandem 5′AGTC repeats and is unique to strain R2846 (starting at nucleotide 1505819; see Table 5). This SSR is found immediately downstream of the presumptive start codon of an ORF encoding a 294 aa protein with homology to the glycosyltransferase 2 family of proteins (PFAM PF000535). In *Hi*, phase variable glycosyltransferases are frequently involved in LPS biosynthesis (Hood et al., 1996a). In strain 86-028NP the same glycosyltransferase is replaced with a different gene (NTHi_1053) that has high homology (e value of 1 × 10^−141^, BLASTN) to the phosphoethanolamine transferase gene, *lpt3,* of *Neisseria meningitidis* (Mackinnon et al., 2002). This is the first report of a gene with significant homology to *lpt3* in *Hi*. Both NT*Hi*_1053 and the gene encoding the putative glycosyltransferase have an atypically low G + C content (<30%), suggesting that they have been acquired by horizontal transfer. The finding that multiple, distinct gene insertions have occurred in the same region of the bacterial genome in different strains may indicate that this is a hotspot for recombination.

The second novel tetranucleotide SSR locus contains a 5′CCAA tract associated with a putative glycosyltransferase (gene NTHi_1769 in strain 86-028NP). This SSR is present in all four genomes examined with between 8 and 16 repeat units and constitutes the first example of a 5′CCAA tract that is associated with a gene other than iron utilisation genes in *Hi* (Jin et al., 1996; Morton and Stull, 1999).

### 3.4 Pentanucleotide SSRs identified as potential mediators of phase variation in *Hi*

A total of eight pentanucleotide SSR loci were identified across the four *Hi* strains investigated, six of these were located within ORFs (see supplementary Table 1). The length of the pentanucleotide SSRs ranged from three to twelve units but the majority were of the minimum threshold value of three units. Two of the pentanucleotide SSRs located within ORFs are of particular interest. The first of these pentanucleotide SSRs is associated with the type I modification enzyme, HsdM (the ORF in the Rd KW20 genome (*HI1287*) is truncated due to the repeat), and has previously been implicated in the phase variable expression of this type I restriction-modification gene (Zaleski et al., 2005). The SSRs identified in the four genome study are one, two or four units in length. van Belkum et al. (1997) and van Belkum (1999) described length variation in the region of this pentanucleotide repeat in a survey of 20 *Hi* strains. Zaleski et al*.* (2005) estimated the phase variation rates of the (5′GACGA)4 (4 tandem repeats of the sequence 5′GACGA3′) pentanucleotide repeat at this locus from observations on the degree of bacterial lysis induced by exposure to phage HP1c1. The rates they recorded for a change from four to three pentanucleotide repeats in strain RM118 were high and equivalent to those previously measured for much longer tetranucleotide repeat tracts (De Bolle et al., 2000) in the same strain.

The second coding pentanucleotide SSR of interest (5′TCAGC) was found in a gene of the *hmg* locus that encodes a high molecular weight glycoform of the LPS (Hood et al., 2004). The two repeat unit pentanucleotide SSRs present in Rd KW20 and R2846 (within the ORFs *HI0867* and *Hflu103000281*, respectively) are consistent with the expression of a putative LPS flippase, whilst the three unit SSR in R2866 is inconsistent with expression of this gene. It is noteworthy that these two potential phase variation-mediating pentanucleotide SSRs relate to gene functions (restriction-modification and LPS modification) whose expression has previously been reported to be phase varied by tetranucleotide SSRs*.*

### 3.5 Heptanucleotide SSRs as mediators of phase variation in *Hi*

Four heptanucleotide SSRs were found in the survey of the four *Hi* genomes, three of which we have designated as potential mediators of phase variation. Two of these heptanucleotide SSRs are located approximately 100 bp upstream of the *hmw1a* and *hmw2a* genes and have previously been described by Dawid et al*.* (1999). They reported that these SSRs are within the promoters of the *hmw1a* and *hmw2a* genes and that alteration of the number of repeat units present in these SSRs results in a modulation of gene expression. The exact mechanism by which these SSRs influence transcription from these genes remains to be determined but may involve modulation of transcription from two alternative start sites (Dawid et al., 1999). Strain R2846 has (5′TGAAAGA)17 and (5′TGAAAGA)16 for *hmw1a* and *hmw2a*, respectively, and strain 86-028NP has (5′TGAAAGA)17 and (5′TGAAAGA)23 units for *hmw1a* and *hmw2a*, respectively, but there are no equivalent loci or repeat tracts in the other two genomes.

The third heptanucleotide SSR with a potential to mediate phase variation is the (5′AACAACC)1-7 tract situated only 13 bp upstream of a gene encoding a member of the TonB-dependent receptor family (PF0593) that has similarity to Fe transport proteins. One unit of the repeat is found in the genomes of strains Rd KW20 and R2846, seven in R2866 and six in 86-028NP. Rd KW20 and R2866 appear to have full length ORFs but the 86-028NP ORF is disrupted by a frameshift unrelated to the SSR. The observed variation in the length of this SSR, together with its position so close to the start of the downstream ORF, led us to postulate that it may mediate phase variation in *Hi*.

### 3.6 Other types of SSRs identified in the *Hi* four genomes study are not considered to mediate phase variable gene expression

Analysis of mononucleotide, dinucleotide, trinucleotide and hexanucleotide SSRs in the four genome study did not provide any evidence to suggest to us that these classes of repeat were associated with phase variable gene expression as detailed below.

### 3.7 Mononucleotide SSRs in the four *Hi* genome are predominantly short A or T tracts

Mononucleotide SSRs have previously been documented as important mediators of phase variation in species such as *Neisseria meningitidis* (Schoen et al., 2007), *Bordetella pertussis* (Gogol et al., 2007) and *Campylobacter jejuni* (Hofreuter et al., 2006; Pearson et al., 2007). Perhaps surprisingly, they have not been implicated in phase variation in *Hi*, although partial sequencing of the *iga* gene from some *Hi* biogroup aegyptius strains led the investigators to suggest that a G10 tract found in only one strain may have mediated phase variable expression of the gene (Kilian et al., 2002).

Our analysis of the mononucleotide SSR loci present in the four *Hi* genomes revealed a considerable degree of heterogeneity in this class of SSR between these strains. 64 homopolymeric tracts were identified across the four genomes and Supplementary Table 1 summarises their characteristics. 28/64 (44%) of the mononucleotide SSRs were found within ORFs and although variations were frequently observed between strains they were not consistent with mediating phase variation (see Supplementary Table 1). The findings from the genome of strain Rd KW20 were representative of the distribution of mononucleotide SSRs found in the three other strains. All of the mononucleotide SSRs in this strain were A or T tracts (18/18) and most were the minimum threshold length of 9 units in length (16/18). Comparison across the four strains revealed that the variation observed in the equivalent mononucleotide SSRs of 8–10 units usually occurred by the substitution of one of the bases within the homopolymeric tract with a different base (e.g. an (A)9 tract was found as (A)7CA in some strains). All substitutions interrupting the A or T homopolymeric SSRs were found to be G or C nucleotides, suggesting an uneven pattern of mutation.

Examination of the further three genomes identified some anomalous mononucleotide SSRs. The first is an exceptionally long (A)34 tract identified in strain R2866. This SSR was located 120 bp upstream of the start of the ORF encoding the autotransporter adhesin Hia, which is an autotransporter protein containing the YadA domain and is believed to bind vitronectin and aid survival in human serum (Cotter et al., 2005a; Hallström et al., 2006; Meng et al., 2006). This SSR is not obviously associated with a promoter region and its function, if any, remains unclear. The second and third are a (G)12 and a (C)11 repeat tract both found in the genome of strain 86-028NP, and which are noteworthy because mononucleotide SSRs of G or C residues are uncommon in *Hi*, reflecting the low G + C content of this organism (38%). The (G)12 SSR was within the 5′ end of ORF *ntHI0694*. This gene shows homology with genes encoding methyltransferases of the FkbM family, some of which are involved in the biosynthesis of methylated sugars in *Rhizobium etli* LPS (Duelli et al., 2001). This gene has not been identified in other *Hi* strains and suggests that 86-028NP LPS may be O-methylated. The (C)11 SSR was located 230 bp upstream of the *acpP* gene (*ntHI0243*). Members of the AcpP family are short proteins which are involved in the transfer of acyl groups and are considered house keeping proteins. In the three other genomes the tract at the same location contains five C residues.

### 3.8 Dinucleotide SSRs in the *Hi* four genome study

Phase variation mediated by dinucleotide repeats has been documented previously in *Hi*. A (TA)9-11 tract, located in the promoter region of two divergently transcribed genes, *hifA* and *hifB* was shown to control fimbriae biogenesis in some strains (van Ham et al., 1993). The *hif* locus is present in only 20% of NT*Hi* strains and, of the four strains analysed here, only R2866 contains the *hifA* and *hifB* genes. In this strain, however, the 5′TA tract was present as a 5′(TA)4ATTA sequence. The threshold value set for dinucleotide SSRs in this study was five, therefore this tract was not identified as an SSR; further discussion of this locus is found later in this paper.

Eight dinucleotide SSR loci were identified in this four genome analysis, all of which were found to be of the threshold value of five repeat units in length. Five were located within coding regions. In a similar fashion to the variation observed for many of the mononucleotide SSRs, seven of the dinucleotide SSR loci were found to have sequence variations that did not alter the overall length of the sequence between strains and so would not cause frameshifts consistent with phase variation. For example, a (CA)5 repeat conserved in the genomes of strains Rd KW20, R2846 and 86-028NP was found to be replaced with CACG(CA)3 in strain R2866.

### 3.9 Trinucleotide SSRs were predominantly found to be located within ORFs

Eighteen trinucleotide SSRs were identified in this study, the majority of which, (15/18), were located within coding regions. All of these 15 SSRs consisted of no more than four repeat units and where variation in the repeats was observed between strains, it either resulted in a reduction in length of the SSR or disruption of the sequence whilst maintaining the same length. The three trinucleotide SSRs that were found in non-coding regions showed greater variation in overall length but were not within identified promoter or other regulatory regions.

### 3.10 Hexanucleotide SSRs identified in the four genome study

Ten hexanucleotide SSRs were identified within the four genomes, eight within ORFs and two in non-coding regions. Variation in coding hexanucleotide repeats can lead to altered amino acid sequence but not phase variable gene expression. The coding region hexanucleotide SSRs identified in this study were either conserved or, like the mononucleotide and dinucleotide variations discussed above, showed changes in sequence but not length and thus were inconsistent with modulating phase variable expression. Of the two non-coding region associated hexanucleotide repeats, one is conserved across all four strains and is present downstream of the closest ORFs, whilst the other 5′TTAAAA SSR is present as three repeat units in Rd KW20, two units in 86-028NP and as two units plus an interrupted third repeat unit in R2866 and R2846. This SSR is situated 19 bp from the start codon of *HI0525* in strain Rd KW20, which encodes a phosphoglycerate kinase involved in central metabolism and the influence of this SSR on the expression of this ORF is unknown.

### 3.11 SSRs with repeat units greater than 7 nucleotides are not found at high frequency in the four genomes

Of the limited number of SSRs with repeat unit lengths greater than seven nucleotides that were identified in the four genomes study, most were found in only one strain. These include a nonanucleotide SSR found in the genome of strain 86-028NP. This (5′GTTTTCTTA)19 SSR was found to be located 92 bp upstream of the *hmw2C* gene. As discussed above, variations in heptanucleotide SSR associated with the *hmw2A* loci are thought to modulate gene expression but the function of this nonanucleotide SSR is not known. An octanucleotide (5′ATTATTTG) SSR however, was found in multiple strains, varying in length between 1 and 6 repeat units. It was found to be located between the divergently transcribed *cmkB* and *pdxS* genes which encode a cytidylate kinase 2 and a pyridoxal biosynthesis lyase, respectively, (designated *HI1646* and *HI1647* in strain Rd KW20). They are both suggested to play roles in metabolism and so it is uncertain whether this SSR would actually be utilised in modulating their expression.

### 3.12 Analysis of the SSRs of a further 12 genomes

Whilst the SSR analysis of the four *Hi* genomes was ongoing, 12 further *Hi* genomes were sequenced and the resulting full or partial sequences made publicly available (listed in Table 1). These 12 additional genome sequences offered us the chance to confirm and extend our detailed SSR analysis of the four *Hi* genomes.

Using the same SSR search methods and threshold values described for the four genome study, 765 SSRs were identified in these 12 additional genomes (summarised in Table 6). From these data it was seen that mononucleotide SSRs are found in 10 out of 12 of the additional genomes at a higher frequency than was observed in the four genome study. However, it should be noted that the 454 sequencing technology used to generate the majority of the further genome sequences has a decreased fidelity for mononucleotide tracts which may account, to some extent, for the higher number of mononucleotides SSR detected in these strains. However, the F3031 and F3043 genomes, for which the highest number of mononucleotide SSRs were identified, were sequenced using ABI Sanger dideoxy sequencing technology.

In a high proportion of the additional genomes, tetranucleotide and hexanucleotide SSR were also observed more frequently than in the four genome study.

### 3.13 Tetranucleotide SSRs in the complete genome collection

The nine NT*Hi* genomes, sequenced by the Center for Genomic Sciences (Hogg et al., 2007) (see Table 1), and the genome of strain 10810, contained a similar number of tetranucleotide SSRs to that previously observed in the four genome study (12-14 per genome) and only two novel tetranucleotide SSR loci were identified. Conversely, in the genomes of strains F3031 and F3043, 18 and 21 tetranucleotide SSR loci were identified, respectively, and eight of these tetranucleotide SSRs were not identified in any of the previously analysed genomes (see Table 5).

### 3.14 Ten novel tetranucleotide SSRs

A total of ten novel tetranucleotide SSR loci were identified in the additional twelve genomes. One locus, *licA2* is a duplication of the *licA* locus reported in the four genome study (Fox et al., 2008) Five of the novel tetranucleotide SSR were associated with genes encoding members of the trimeric autotransporter protein family which commonly contain a C-terminal YadA domain (PFAM03895) (Cotter et al., 2005b; Koretke et al., 2006). All five of these paralogous loci were present in the two *Hi* biogroup aegyptius strains F3031 and F3043 and one of the loci was also present in the genome of the NT*Hi* strain, PittHH. Previously described members of this family of proteins from *Hi* include the adhesins Hsf and Hia which have been implicated in virulence (Cotter et al., 2005a; Surana et al., 2004). It can be envisaged that the expression of adhesins may not be advantageous in all growth conditions as they are possible targets for the host immune system and are large proteins (up to 1016 aa) whose expression would require considerable resources. Indeed, the NadA protein from *N. meningitidis* which is a member of this family of proteins, has previously been shown to be phase variably expressed (Capecchi et al., 2005; Martin et al., 2005). *Hi* biogroup aegyptius strains have been associated with atypical invasive disease and it is, therefore, tempting to speculate that the high number of putative phase variable adhesins identified in strains F3031 and F3043 may somehow contribute to the unusual clinical outcomes associated with these strains.

An additional four novel tetranucleotide SSRs were identified from strains F3031 and/or F3043. The first, a (5′ATTA)9 SSR is found 225 bp upstream of a gene encoding a putative DNA repair enzyme, formamidopyrimidine-DNA glycosylase MutM, in strain F3043. The equivalent position in other *Hi* strains contains 3 copies of the 5′ATTA repeat unit. The role of this repeat in expression of MutM is unknown but variations in the expression of *mutM* could potentially result in altered mutation rates in *Hi* (Horst et al., 1999).

The second novel tetranucleotide SSR identified from strains F3031 and F3043 is a 5′CAAT SSR contained within the 5′ region of an ORF that encodes a putative glycosyltransferase with homologies to glycosyltransferase family 8 (PFAM01501). Homologues of this gene are found in other strains of *Hi* (including *HI0223* in strain Rd KW20) but without the associated SSR. The function of this gene is unknown but it may contribute to LPS expression in strains F3031 and F3043.

The third of the four additional novel tetranucleotide SSR loci contains (5′CAAT)21 and was found only in strain F3043. It is located within the 5′ end of an ORF that encodes a putative S-adenosylmethionine (SAM)-dependent methyltransferase and shows some homology to HI0096 in strain Rd KW20. SAM-dependent methyltransferases have been implicated in various cellular processes including protein trafficking and sorting, signal transduction, biosynthesis, metabolism, and gene expression.

The final novel tetranucleotide SSR identified in strain F3031 is a (5′CAAG)32 SSR located 58 bp upstream of a gene encoding an adenine specific methylase homologue (EcoRI) and 202 bp upstream of the divergently transcribed *htpX* (which encodes a putative protease protein, induced by heat shock in *E. coli*). HtpX has not been investigated in *Hi* but in *E. coli* it is part of the membrane-localised proteolytic system and may play a part in the degradation of unstable membrane proteins (Sakoh et al., 2005).

### 3.15 Consideration of characteristics of tetranucleotide repeats from the complete genome collection

**Fig. 1:**
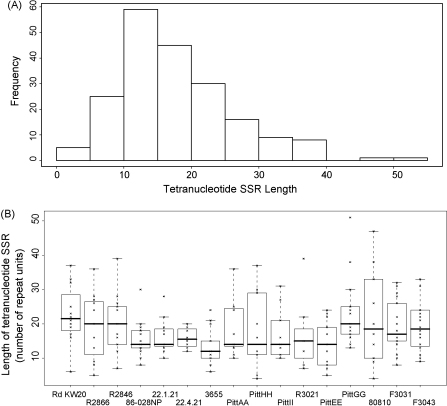
Histogram and boxplot representations of the length distribution, sequence, strain and loci associations of tetranucleotide SSRs. (A) Frequency histogram of tetranucleotide SSR length distribution in the complete genome study. (B) Boxplot analysis of the relationship between the strain from which the genome was derived and the length of the tetranucleotide SSRs.

In total, 199 tetranucleotide SSRs associated with 28 different loci and consisting of nine different repeat unit sequences have been identified in the complete genome collection. The distribution of tetranucleotide SSR length, and the relationship between length of tetranucleotide SSR and strain are shown in Fig. 1. The length of an individual tetranucleotide SSR does not appear to be dependent on strain background, repeat unit sequence or locus (Fig. 1B), and a wide degree of variation and considerable overlap between groupings is observed. Fig. 1A shows that despite differences in the source, date of isolation and associated clinical symptoms of the different strains there is an approximately normal distribution of tetranucleotide SSR lengths. Fig. 1B shows that the two *Hi* biogroup aegyptius strains F3031 and F3043, which are associated with unusual clinical symptoms and have the highest number of tetranucleotide SSR, display a similar distribution of SSR lengths to all other strains.

### 3.16 Consequences of the sequence and location of tetranucleotide SSR

As noted previously, the tetranucleotide SSRs identified in the four genome study of *Hi* are located within ORFs and, with only two exceptions, are located immediately adjacent to or just downstream of the translational start site. In this position, any frameshift due to variation in length of the SSR, would result in a peptide being made from the incorrect reading frame and a premature stop to translation. The location of tetranucleotide SSRs within the 5′ region of the ORFs limits the encoded tetrapeptide repeat to the N-terminus of the respective protein. The two exceptions to this pattern are the 5′GCAA tetranucleotide SSR located in the middle of the *oafA* gene that has been previously described (Fox et al., 2005), and a 5′GACA tetranucleotide SSR located in the 3′ region of a gene encoding a putative glycosyltransferase (*pgt1*) in the genomes of strains R2846, 86-028NP, 3655 and PittEE. These repeats may modulate the protein function rather than control ON/OFF switching of its expression.

Tetranucleotide SSRs may constitute a substantial proportion of the coding region of a gene and thus the repeat unit sequence will have a significant influence on the amino acid composition of the encoded protein. The constraints that this may impose, in terms of permissible tetranucleotide sequences, have not been well characterised although High et al. (1996) suggest that the peptides encoded by the repeat regions form structurally flexible regions that loop out of the protein structure and therefore do not interfere with tertiary structure.

An *in silico* analysis of the repeat sequences identified in the 16 *Hi* genomes analysed was performed and hydrophilic amino acids are over represented in the SSR encoded peptides, compared with their frequency in the normal proteome. Of the eight tetranucleotide repeat sequences found within ORFs in *Hi*, five encode hydrophilic peptides with no net charge (5′CAAT, 5′GACA, 5′CCAA, 5′AGCC, and 5′AGTC), one encodes a hydrophobic peptide with no net charge (5′TTTA) and two encode hydrophilic peptides with a net positive charge (5′GCAA and 5′CAAA). The high proportion of hydrophilic peptides encoded by the tetranucleotide SSRs and their frequent N-terminal location suggests that they are likely to be surface exposed and have the opportunity to ‘loop out’ of the folded protein structure and thus be less likely to interfere with the tertiary structure of the protein and, therefore, its function. The exception is a 5′TTTA tetranucleotide SSR which encodes a hydrophobic peptide within a putative drug/metabolite exporter (*HI0687* in strain Rd KW20). Transmembrane helices predictions (TMHMM server v2.0, Sonnhammer et al., 1998) suggest that the portion of this protein encoded by the SSR lies entirely within a transmembrane domain. Examination of homologues of the *HI0687* gene indicates that the hydrophobic nature of such transmembrane helices is well conserved but often the primary sequence is not (data not shown). Another observation of this study was that although previously SSRs of a particular tetranucleotide repeat unit sequence have been associated with genes of related function, e.g. 5′CCAA tracts with genes encoding iron utilisation proteins (Jin et al., 1996; Morton and Stull, 1999), in this study, we have found no evidence of a particular tetranucleotide repeat unit sequence being restricted to a particular class of gene.

### 3.17 Interrupted tetranucleotide SSR may be an indication of intra-genome recombination between paralogous loci

One feature of certain tetranucleotide SSR, noted during the course of this study was their interruption by an imperfect repeat unit. All of the genomes in this study were found to contain between one and four related, hemoglobin/hemoglobin–haptoglobin-binding (*hgp*) genes containing 5′CCAA SSR that show considerable variation in length. *Hi* lacks most of the genes of the heme biosynthetic pathway and requires hemoglobin/hemoglobin–haptoglobin-binding proteins to capture heme-containing compounds required for growth (Morton and Stull, 1999). Seven interrupted tetranucleotide SSRs were observed in total in this analysis of which six were found to be associated with *hgp* genes. We postulate that homologous recombination occurring between these paralogous loci may occasionally generate imperfect repeats and it will be of interest to ascertain whether similar events occur between other duplicated loci, e.g. paralogous adhesin genes (discussed below), partial or fully duplicated *hifA* loci and duplicated *lic1A* genes (strain PittGG).

### 3.18 Mononucleotides identified as potential mediators of phase variation in the analysis of the 12 further genomes

In contrast to the four genome study, the analysis of the 12 additional *Hi* genomes has identified a number of mononucleotide SSRs with the potential to mediate phase variable gene expression. These mononucleotide SSRs were located within the 5′ coding regions of ORFs, associated with frameshift mutations, or located within potential promoter regions. The potential phase variable genes include those encoding virulence-related factors such as glycosyltransferases, type-I restriction modification systems, haemagglutinins, YadA domain containing proteins, pilin genes and a Fe–S cluster assembly scaffold protein (see Table 7). This study offers the first indication that mononucleotide SSRs may mediate phase variation in *Hi*.

Further support to the role of the mononucleotide SSRs in mediating phase variation in *Hi* is that some genes identified in the 12 genome study have previously been determined to be phase variable but mediated by other classes of SSRs. An example is the divergently transcribed pilin genes, *hifA* and *hifB,* which Geluk et al. (1998) demonstrated to be phase variable due to variation in the length of a 5′TA SSR located between them and 104–225 bp upstream of the *hifA* gene. Changes in the length of the dinucleotide SSR were proposed to alter the spacing between the −10 and −35 promoter sequences and therefore alter expression of the genes. In strain F3031, there are four *hifA* loci in total. Two of the loci have an arrangement similar to that described by Geluk et al. (1998) with the 5′TA SSR located between the divergently transcribed *hifA* and *hifB* genes whilst the other two *hifA* loci have mononucleotide (A17 or A12) instead of dinucleotide SSRs located either 63 or 93 bp upstream of *hifA* (see Table 7)*.* There is no *hifB* gene associated with these latter loci. Phase variation of pilin expression mediated by mononucleotide SSRs has not been previously reported in *Hi*. The exact location and extent of the promoter region of *hifA* has not been mapped in these strains, but the position of the mononucleotide SSRs makes them a candidate to mediate phase variation.

In the four genome study, homopolymeric A or T tracts of less than 11 bp were found with only one exception, an A34 tract found in the R2866 genome. In the additional twelve genomes a similar tract of between 20 and 49 bp was found in four strains in the same genomic location; approximately 120 bp upstream of the nearest ORF which encodes a protein with homology to YadA-domain containing proteins such as Hsf. The function of this SSR is unknown but it is tempting to speculate that it may play a role in regulating the expression of the downstream Hsf-like encoded protein. Similarly, in the genome of strain F3031 the expression of a number of YadA domain containing proteins was suggested to be mediated by tetranucleotide SSRs (see Table 5). However in one instance, the expression of a YadA domain containing protein in this strain is potentially mediated by a G13 SSR (located at base 548152) located 10 bp within the ORF (see Table 7). The association of mononucleotide SSRs, in certain strains, with paralogs of genes which are phase variable by other SSRs offers strong circumstantial evidence that these mononucleotide SSRs may mediate phase variation in *Hi*.

### 3.19 Other potentially phase variable SSRs in the complete genome collection

The heptanucleotide SSRs associated with the *hmw1a* and *hmw2a* genes in the four genome study were also identified in the genomes of strains PittEE and R3655 in the 12 further *Hi* genomes analysed. In PittEE, 13 copies of the heptanucleotide repeat are present 69 bp upstream and 38 copies 104 bp upstream of the *hmw1a* and *hmw2a* genes, respectively, and in R3655, 16 copies of the repeat are present 106 bp upstream of *hmw1a*. However, in the further genome study, an additional novel heptanucleotide SSR associated with *hmw1a* was identified in the genome of strain PittAA. Interestingly, this SSR, consisting of (5′AATTTTG)14, was 3.5 kb within the 7.3 kb putative full length ORF rather than in the promoter region, and a frame shift had occurred which is consistent with this being caused by variation in the length of the SSR. In the further genome analysis, an octanucleotide SSR was found associated with *hmw* loci. This SSR contained twelve to fifteen copies of a 5′GCATCATC repeat and was identified 200–213 nucleotides upstream of the *hmw1a* and *hmw2a* loci of strain F3043 and F3031.

A further novel heptanucleotide SSR with the potential to mediate phase variation was identified in strains 22.4.21 and R3655, within an ORF which is a homologue of the *HI1369* gene (encoding a putative TonB dependent iron ligand gated channel). Thirteen units of the 5′AACAACC repeat are found in 22.4.21, and eight repeat units in strain R3655 which results in a truncated ORF due to a frameshift.

An octanucleotide SSR identified in the four genome study as containing one, four or six copies of a 5′ATTATTTG unit 12 bp upstream of a gene encoding a pyridoxine biosynthesis protein, was also identified in seven strains of the further twelve genome collection (four copies of the SSR in strains PittHH, PittAA and 22.1.21 and six copies in strains R3655, PittII, F3043 and Hib; see Table 7). However, the limited range of variation and relatively short length of this SSR are not what would be expected at a classically phase variable locus and so the significance of this SSR at this location remains uncertain.

## 4 Discussion

The complete genome sequence of *Hi*, strain Rd KW20 (Fleischmann et al., 1995), provided for the first time the means to analyse the gene content, organisation and sequence structure of a free-living organism. One of the major findings in *Hi* strain Rd KW20 was the association of SSRs, especially tetranucleotide SSRs, with genes involved in host adaptation, commensalism and virulence (Hood et al., 1996b). SSRs are hypermutable and mediate a high frequency of reversible increases or decreases in the number of repeat units resulting in phase variable expression of the associated genes (Moxon et al., 2006).

As sequencing techniques have progressed, the ease with which sequencing data can be gathered has increased. As a result, the sequences of multiple strains of a single species have become available for comparison and the extent of genomic variation between strains has become evident. In this study, our aim was to extend our understanding of the role of SSRs in the biology and pathogenicity of *Hi* by an analysis of four complete genome sequences and a survey of available sequence data for a further twelve strains.

SSRs, consisting of repeat units of between one and nine nucleotides in length, were characterised. For this analysis to be practical, it was necessary to establish threshold values, above which tandem repeat units were designated as SSRs. Data pertaining to the genomic location, position relative to the nearest ORF and the types of polymorphism observed by comparison between genomes was compiled for each of 223 SSRs in the initial survey of the four complete *Hi* genome sequences from strains Rd KW20, R2846, R2866 and 86-028NP. These SSRs were broadly classified into three categories; invariant, variant and potentially phase variable. Invariant SSRs showed no variation in sequence, position or length between strains whilst variant SSRs showed some variation between strains but not of a type that would mediate phase variation, i.e. they usually showed some variation in sequence but not overall length. Potentially phase variable SSRs showed variation in the number of repeat units constituting the SSR between strains and were in positions consistent with mediating phase variation either within ORFs or promoter regions. The majority of SSRs examined fell into the first two classes. From the further 12 partial and complete genome sequences, 765 additional SSRs were identified.

These studies have confirmed that tetranucleotides are the predominant class of SSR to mediate phenotypic variation via phase variation in *Hi*. A total of 199 tetranucleotide SSRs were found distributed across the 16 strains, associated with 28 different loci (see Table 5). Of these, 10 were novel tetranucleotides, eight of which were identified in the genome sequences of only two strains, the *Hi* biogroup aegyptius strains F3031 and F3043. Tetranucleotide SSRs were found associated with a number of paralogous adhesin genes in these strains and, intriguingly, with a *mutM* locus that could potentially modulate mutation rates due to oxidative damage (Horst et al., 1999).

The *Hi* biogroup aegyptius strains, F3043 and F3031 isolated in Brazil, were associated with conjunctivitis and BPF, respectively. A relevant question is whether the increased number of tetranucleotide SSRs in these strains may contribute to their unusual virulence phenotype. A detailed analysis of the characteristics of the tetranucleotide SSRs across all strains showed that whilst the number of tetranucleotide SSRs was higher in the biogroup aegyptius strains (Table 6), the length or sequence of the SSRs was similar between all the strains (Fig. 1 and unpublished data). Indeed, no relationship was found between the sequence, length, genomic locus or protein function of tetranucleotide SSRs. Other tetranucleotide SSR loci identified included those encoding two glycosyltransferases, one of which contains a 5′CCAA repeat, the first occasion for *Hi* where this particular SSR unit sequence has been associated with genes encoding proteins of any function other than hemoglobin and hemoglobin–haptoglobin binding.

Although tetranucleotide SSRs are the most frequent mediators of phase variation in *Hi*, other SSRs may play a role in mediating phase variation, particularly in strains such as F3031 and F3043. This study has identified a number of novel mononucleotide, dinucleotide, pentanucleotide, heptanucleotide, and octanucleotide SSRs as potential mediators of phase variation. Mononucleotide SSRs have not previously been described as frequent mediators of phase variation in *Hi*, in contrast to other bacterial species such as *N. meningitidis*. There is only one report in the literature of mononucleotide SSRs potentially mediating phase variation in *Hi*; a G10 SSR is suspected to mediate phase variation of the *iga* gene, AF522258, in *Hi* biogroup aegyptius strain HK266 (Kilian et al., 2002). However, the distribution of the mononucleotide SSR loci identified in this study suggests that there may be some strain-dependent differences in the use of mononucleotide SSR to mediate phase variation. The potential mononucleotide SSR-mediated phase variable genes identified include those encoding factors associated with virulence such as glycosyltransferases, type-I restriction modification systems, haemagglutinins, YadA domain containing proteins (Cotter et al., 2005b; Koretke et al., 2006), pilin and a Fe–S cluster assembly scaffold protein (see Table 7). A number of the genes where phase variation is potentially mediated by homopolymeric tracts are phase variable by other mechanisms in other strains. For example, *hifA* and *hifB* expression is usually mediated by a dinucleotide SSR (van Ham et al., 1993). Similarly, the expression of YadA-domain containing proteins is potentially mediated by tetranucleotide SSRs in some loci identified in this study and by mononucleotide SSRs in other loci whilst the expression of the *hmw1A* and *hmw2A* genes is potentially mediated by upstream heptanucleotide, octanucleotide or nonanucleotide SSRs in different strains.

Differences in the classes of SSRs which mediate phase variation between species, or even different strains of one species, may be determined by inter species/inter strain differences in DNA metabolism as the efficiency with which different types of slippage intermediates are recognised and repaired is reliant upon the complement of DNA repair mechanisms in the given strain/species. Investigation of the molecular basis of these differences will be aided by the availability of full genome sequences in conjunction with experimental assays.

The strains examined in this study were isolated in the United Kingdom (one strain), Brazil (two strains) and the United States of America (13 strains). A majority of the novel SSRs identified were in the F3031 and F3043 genome sequences (the Brazilian strains) and it remains unknown whether the population/geographical structure of *Hi* strains may be a significant factor in determining the complement of SSR within a strain: until the population structure of *Hi* is better understood it is difficult to predict the size of the SSR pan-genome and its potential role in mediating phase variation. In the strains studied, with the exception of F3031 and F3043, there were no associations between the ability to cause disease or commensal infection in the strains and the complement of potential phase variable mediating SSRs. For each strain, the contribution of the number and complement of phase variable genes to the probability of pathogenic potential remains unknown.

In conclusion, this study has reaffirmed the primacy of tetranucleotide SSRs as mediators of phase variation in *Hi* and has characterised and compared 28 tetranucleotide SSR loci (9 of them previously unreported) across 16 strains. Additionally, this study has identified a number of previously unrecognised mononucleotide, dinucleotide, pentanucleotide, heptanucleotide, and octanucleotide SSRs as potential mediators of phase variation that will be the focus of future research efforts. Thus, the utility of whole genome sequences in the investigation of the biology of pathogenic bacteria has been confirmed and, further, the analysis of multiple genomes has revealed non-intuitive subtleties in the population structure concerning the distribution of SSRs across the *Hi* pan-genome.

## Figures and Tables

**Table 1:** Characteristics of genome sequences used in this study.

Strain	GenBank accession number	Source strain information^a^	Reference^b^
Four genome study			
Rd KW20	NC_000907	Acapsulate serotype d, nasopharynx, USA	Fleischmann et al. (1995)
86-028NP	NC_007146	NT*Hi*, isolated from a child with otitis media, USA	Harrison et al. (2005)
R2846^c^		NT*Hi*, middle ear fluid of a child with acute otitis media	http://www.genome.washington.edu/UWGC/Hinf/index.cfm (strain 12, Barenkamp and Leininger, 1992)
R2866^c^		NT*Hi*, blood of a child with meningitis	http://www.genome.washington.edu/UWGC/Hinf/index.cfm (Int1, Nizet et al., 1996)

Twelve genome study			
PittGG	NC_009567	NT*Hi*, external ear discharge from otorrhea, USA	Hogg et al. (2007)
PittEE	NC_009566	NT*Hi*, chronic otitis media with effusion, USA	Hogg et al. (2007)
R3021^d^	NZ_AAZJ00000000	NT*Hi*, nasopharynx of a healthy individual, USA	Hogg et al. (2007)
PittII^d^	NZ_AAZI00000000	NT*Hi*, chronic otitis media with effusion, USA	Hogg et al. (2007)
PittHH^d^	NZ_AAZH00000000	NT*Hi*, chronic otitis media with effusion, USA	Hogg et al. (2007)
PittAA^e^	NZ_AAZG00000000	NT*Hi*, middle ear effusion of a child with chronic otitis media, USA	Hogg et al. (2007)
3655^d^	NZ_AAZF00000000	NT*Hi*, middle ear effusion of a child in Missouri with acute otitis media, USA	Hogg et al. (2007)
22.4.21^d^	NZ_AAZE00000000	NT*Hi*, nasopharynx of a healthy individual, USA	Hogg et al. (2007)
22.1.21^d^	NZ_AAZD00000000	NT*Hi*, nasopharynx of a healthy individual, USA	Hogg et al. (2007)
F3031^d^^,^^e^	NZ_AADO00000000	*Hi* biogroup aegyptius, Brazilian purpuric fever, Brazil	http://www.sanger.ac.uk/sequencing/Haemophilus/influenzae/F3031/
F3043^d^^,^^e^	NZ_AADP00000000	*Hi* biogroup aegyptius, purulent conjunctivitis, Brazil	http://www.sanger.ac.uk/sequencing/Haemophilus/influenzae/F3043/
10810		Serotype b, meningitis isolate, UK	http://www.sanger.ac.uk/Projects/H_influenzae/

The four strains for which the complete genome sequences were available at the commencement of this study are listed in the upper portion of the table; the further twelve strains for which full or partial sequences were later acquired are detailed in the lower portion.

a Lists the serotype, the site of isolation and associated clinical features, and country of isolation.

b Where there is no appropriate publication available, the www address from which sequences were obtained is listed.

c The complete genome sequences for these strains were made available to us courtesy of Dr Alice Erwin.

d Incomplete genomes.

e The genome sequences for these strains were made available to us courtesy of Prof. Simon Kroll and the Welcome Trust Sanger Sequencing Centre.

**Table 2:** Determination of the minimum threshold for SSR detection in *H. influenzae* genome sequences, with reference to strain Rd KW20.

Repeat unit length		Threshold value^a^	Maximum expected length^b^	Number of SSRs in Strain Rd-KW20 Genome identified using^c^		
No.	Prefix			Threshold value −1	Threshold value	Threshold value +1
1	Mono	9	13	163	18	2
2	Di	5	5	88	5	0
3	Tri	4	4	814	13	1
4	Tetra	3	3	38	12	12
5	Penta	3	2	1676	5	2
6	Hexa	3	ND	940	5	1
7	Hepta	3	ND	126	0	0
8	Octa	3	ND	45	0	0
9	Nona	3	ND	34	2	1

				3924	60	19

This table sets out the minimum number of repeat units (threshold value) for the identification of SSRs of each repeat unit length used in this study. ND: not determined.

a The threshold value is the minimum number of repeat units required to be present in an uninterrupted tandem arrangement in order for that sequence to be defined as an SSR.

b Maximum expected length is the maximum number of repeat units of each designated length that would be expected to occur in the Rd KW20 genome as predicted by hidden Markov model analysis (Paul Swift, personal communication).

c The values in columns Threshold value −1 and Threshold value +1, show the number of SSR identified of each motif length in the Rd KW20 genome if the threshold value is decreased or increased by one unit, respectively.

**Table 3:** The frequency and location of SSRs in the genome sequences of four *H. influenzae* strains.

Repeat unit length^a^	Threshold value^b^	Genome^c^			
		86-028NP	Rd KW20	R2846	R2866
1	9	**16** (7)	**18** (7)	**13** (5)	**17** (8)
2	5	**6** (4)	**5** (3)	**4** (4)	**5** (3)
3	4	**8** (6)	**13** (11)	**11** (10)	**9** (7)
4	4	**14** (14)	**12** (12)	**13** (13)	**12** (12)
5	3	**3** (2)	**5** (4)	**3** (3)	**3** (3)
6	3	**4** (3)	**5** (4)	**3** (3)	**3** (3)
7	3	**3** (0)	**0** (0)	**4** (1)	**2** (0)
8	3	**1** (0)	**0** (0)	**1** (0)	**1** (0)
9	3	**1** (0)	**2** (0)	**1** (1)	**2** (1)

Total		**56** (36)	**60** (40)	**53** (39)	**54** (37)

The frequency of SSRs, of each repeat motif length, is given for each of the four genomes in the four genome analysis.

a Repeat unit length: the number of nucleotides that compose a single repeat unit.

b Threshold value is the minimum number of repeat units required to be present in an uninterrupted tandem arrangement in order for that sequence to be defined as an SSR.

c Numbers in bold indicate the number of each type of SSR within each genome; numbers in parentheses show the number of each type of SSR located within predicted ORFs for each genome.

**Table 4:** Assessment of SSR variability and potential to mediate phase variation in the four genome study.

Repeat unit length	Repeat type categories^a^		
	Potentially phase variable	Variable	Invariable
1	**0** (0)	**24** (7)	**14** (7)
2	**0** (0)	**7** (4)	**1** (1)
3	**0** (0)	**12** (9)	**6** (6)
4	**14** (14)	**0** (0)	**4** (4)
5	**2** (2)	**3** (3)	**3** (1)
6	**0** (0)	**8** (6)	**2** (2)
7	**3** (0)	**2** (1)	**0** (0)
8	**0** (0)	**1** (0)	**1** (0)
9	**0** (0)	**3** (2)	**2** (0)

Each SSR identified in the four genome analysis was manually assessed to determine whether or not it was likely to mediate phase variable gene expression, from consideration of its position within an ORF or relative to adjacent ORFs, together with the variation observed between strains. Numbers in bold indicate the number of each type of SSR within each category; numbers in parentheses show the number of each type of SSR located within predicted ORFs for each category.

a This assessment assigned each SSR to one of three categories: potentially phase variable – SSRs that both varied in length and were located in positions likely to influence gene expression; variable – SSRs that showed differences in length or sequence between strains but not of a manner consistent with mediating phase variable expression, and invariable – SSRs that did not show any variation in length, position or sequence between the strains.

**Table 5:** The sequence and number of repeat units that comprise each of the 199 tetranucleotide SSRs identified in 16 *H. influenzae* genomes.

SSR loci number	Associated ORF or genome location^a^	Description^b^	Repeat Unit Seq.	Strain															
				RdKW20	R2866	R2846	86-026	22.1.21	22.4.21	3655	PittAA	PitlHH	PittII	R3021	PittEE	PittCG	10810	F3031	F3043
1	HI_0258	*igtC* glycosyltransferase	GACA	22	26	20	10	13	12	12	14	12	16	12	8	38	17	8	24
2	HI_0352	*lic3A* lipopolysaccharide sialyltransferase	CAAT	33	20	26	19	19	19	24	36	20	14	31.8^d^	19	20	38	22	22
3	HI_0550	*tic2A* lipopolysaccharide glycosyltransferase	CAAT	23	5	25	14	14	e	12	25	11	II	22	23	25	20	15	14
4	HI_1537	*licA* lipopolysaccharide phosphocholine transferase	CAAT	17	36	7	15	22	18	15	25	37	27	15	24	51	47	26	16
5		*licA2* lipopolysaccharide phosphocholine transferase	CAAT													19		31	17
6	HI_0635	*hgp* hemoglobin and hemoglobin–haptoglobin binding protein	CCAA	21	28	25	20	28	16	8	4.10^d^	4	11	e	8	16	33	29	24
7	HI_0661	*hgp* hemoglobin and hemoglobin–haptoglobin binding protein	CCAA	20	27	39	12			6.6^d^	17		23.8^d^		19	23	9	20	10
8	HI_1565	*hgp* hemoglobin and hemoglobin–haptoglobin binding protein	CCAA	19		28	17			8.12^d^			16		9	30		15	13
9	HI_0712	*hgp* hemoglobin and hemoglobin–haptoglobin binding protein	CCAA	37															
10	HI_0687	Drug/metabolite exporter	TTTA	6													4		
11	HI_1058	*mod* type III restriction/modification system modification methylase	AGCC	(TGAC)_32_^c^	16			14	20			12	27					22	IS
12	Hi_1386US	Putative glycosyltransferase	CCAA	16	8	12	13	10		10			10	7	14		10	10	16
13		Within cryptic *yadA-*like gene and 245 bp upstream of *tolC-*like receptor gene	CAAG	25	13	24	14	17	13		11	30	13	15	17	13	14		
14	r2846v6.916	*pgtl* putative glycosyltransferase	GACA			16	14			6	13				5				
15	r2846v6.1528c	Ipt3 region	AGTC			14				10					14				
16	r2846v6.1683	lex2A	CAAG		24	17	14	18	15	15	24	16	21		22	18	26	14	31
17	r2866v6.124c	*lav* AIDA-I/VirG/PerT family of virulence-associated autotransporters	CAAG		20		30	14					14			17		28	
18	12846 V6.202	*oqfA* O-antigen lipopolysaccharide acetylase	CAAG		9	14	8	12	14	11	14	5	11	8	7	14		11	11
19	NTHI1034	*lic3B* lipopolysaccharide sialyltransferase	CAAT				15			21	10				12	24			
20	PITTII	Gene encoding a YadA domain containing protein	CAAG									20						15	24
21	F3043-1499724	Gene encoding a YadA domain containing protein	CAAG																19
22	F3043-196894	Gene encoding a YadA domain containing protein	CAAA															15	13
23	F3043-756964	225 bp upstream gene encoding a YadA domain containing protein	CAAA															16	31
24	F3043-609747	Glycosyltranslerase (family 8) with framshift	CAAT															18	33
25	F3043-1083776	Gene encoding a YadA domain containing protein	CAAG																23
26	F3043-1500170	SAM-dependent methyltransferase	CAAT																21
27	F3043-1598734	225 bp upstream formamidopynmidine-DNA glycosylase (*mutM*)	ATTA																9
28	F3031-1121634	58 bp upstream adenine specific methylase (EcoRI) and 202 bp upstream of *htpX* (heat shock protein)	CAAG															32	

Listed in this table are the 199 tetranucleotide SSRs that are associated with the 28 loci identified in this analysis of the genomes of 16 *Hi* strains.

a The associated ORF designation in the Rd KW20 genome, or when not present in the Rd KW20 genome the R2486, R2866 or 86-028NP genomes. SSR from unannotated genomes are identified by the strain, a hyphen and the nucleotide number at which they are found.

b Genes associated with the SSR. The gene names and predicted functions are from BLAST similarity searches and the published genome annotations.

c The sequence of the tetranucleotide SSR in this locus is different to that associated with this gene in all other strains (5′AGCC).

d *x*…*y* indicates that there are *x* number of the tetranucleotide repeat unit, followed by an interruption, followed by *y* number of tetranucleotide repeat units.

e The presumptive position of the SSR loci is at the end of a contig and its presence or absence cannot be determined.

**Table 6:** The number of SSRs, of each repeat unit length, in each genome.

Repeat unit length	Range in four genome study^a^	Strains^b^											
		22.1.21	22.4.21	3655	PittAA	PittHH	PittII	R3021	PittEE	PittGG	10810	F3031	F3043
Mono	13–17	**25**	**21**	**22**	**20**	**20**	**20**	**28**	12	**19**	16	**30**	**25**
Di	4–6	6	8	2	3	6	6	5	2	6	4	**7**	5
Tri	8–13	12	13	11	11	6	10	12	13	**14**	12	10	6
Tetra	12–14	*11*	9	**17**	13	**9**	**15**	*8*	14	13	*10*	**18**	**21**
Penta	3–5	4	2	3	4	3	4	2	3	**6**	*2*	**6**	5
Hexa	3–5	**6**	4	2	**6**	**6**	5	4	**7**	5	**8**	**9**	5
Hepta	0–4	2	1	2	4	1	2	2	4	1	0	3	1
Octa	0–1	1	1	2	1	2	1	0	0	0	1	**5**	**5**
Nona	1–2	0	0	2	1	0	0	0	0	**3**	0	0	0

Total	53–60	67	59	63	63	53	63	61	55	67	53	88	73

Analysis of the number of SSRs identified in the 12 genome study compared to the four genome study. Numbers given in bold or italic indicate that the value is higher or lower than the four-genome study range, respectively.

a The minimum and maximum number of each type of SSR observed per genome in the four genome study.

b The number of SSRs per genome, of each repeat unit length, in the 12 genome study.

**Table 7:** Notable non-tetranucleotide SSRs identified in complete *H. influenzae* genome collection.

Genome	Repeat unit length	Repeat unit sequence	Number of repeats^a^	Description of location^c^	Genome location (bp)^b^
F3043	1	A	17	67 bp upstream of *hifA* (pilin)	1039014
F3043	1	A	12	93 bp upstream of *hifA* (pilin)	1207519
F3031	1	G	13	10 bp within putative YadA-domain containing protein encoding gene	548152
F3031	1	G	9	FS 625 bp into putative glycosyltransferase gene	556417
F3031	1	G	9	FS 641 bp into type I restriction-modification system gene	751900
F3031	1	T	12	3 bp upstream of putative YadA-domain containing protein encoding gene	1367593
F3031	1	T	12	93 bp upstream of *hifA* (pilin)	1705139
PittEE	1	C	12	FS within 3′ end of a putative O-methyltransferase encoding gene (31% GC)	10582
PittEE	1	C	9	115 bp within Fe–S cluster assembly scaffold gene	697640
PittEE	1	G	9	324 bp within transferrin-binding protein 2 gene	1384490
r3655	1	G	15	118 bp upstream of exonuclease ABC subunit C	1266118
r3655	1	T	12	upstream hemoglobin-binding protein A encoding protein encoding gene	1338394
r3655	1	G	10	FS 114 bp within Fe–S cluster assembly scaffold protein encoding gene	1547902
PittAA	1	C	9	114 bp within Fe–S cluster assembly scaffold protein encoding gene	1350019
PittAA	1	C	11	454 bp within O-methyltransferase gene	1764602
PittII	1	C	9	within truncated *lic3B*, not in other strains	237746
PittII	1	T	36	120 bp upstream of YadA-domain containing autotransporter adhesin gene	983892
R3021	1	T	49	122 bp upstream of YadA-domain containing autotransporter adhesin gene	701090
22.1.21	1	T	20	121 bp upstream of YadA-domain containing autotransporter adhesin gene	1278422
10810	1	A	38	120 bp upstream of YadA-domain containing autotransporter adhesin gene	1960236
F3031	2	TA	10	225 bp upstream of *hifA*, 121 bp upstream of *hifB* (chaperon)	45074
F3031	2	TA	10	225 bp upstream of *hifA*, 121 bp upstream of *hifB* (chaperon)	150062
F3031	2	TA	8	104 bp upstream of YadA-domain containing protein gene	1258000
22.1.21	2	TA	9	166 bp upstream of *hifA* (pilin), truncated *hifB* (chaperon)	52984
22.4.21	2	TA	9	133 bp upstream of *hifA* (pilin), truncated *hifB* (chaperon)	937926
PittEE	7	TGAAAGA	13	69 bp upstream of *hmw1A*	750553
PittEE	7	TGAAAGA	38	104 bp upstream of *hmw2A*, 154 upstream of *NTHI1451* homolog	1118607
PittAA	7	AATTTTG	14	FS 3.5 kb within *hmw1A*	1861090
PittAA	7	TGAAAGA	16	106 bp upstream of *hmw1A*	876652
r3655	7	AACAACC	8	FS within *HI1369* (Fe ligand_gated_channel, TonB dependent)	763559
22.4.21	7	AACAACC	13	77 within *HI1369* (Fe ligand_gated_channel, TonB dependent)	1296925
F3043	8	ATTATTTG	6	12 bp upstream of pyridoxine biosynthesis protein gene	1003128
F3043	8	GCATCATC	13	213 bp upstream of *hmw*1A	1315267
F3043	8	GCATCATC	12	209 bp upstream of *hmw*2A	192933
F3031	8	GCATCATC	15	200 bp upstream of *hmw*2A	1349049
F3031	8	GCATCATC	14	213 bp upstream of *hmw*1A	1603867
r3655	8	ATTATTTG	6	12 bp upstream of pyridoxine biosynthesis protein (near end of contig)	1203993
PittHH	8	ATTATTTG	4	12 bp upstream of pyridoxine biosynthesis protein gene	1799917
PittAA	8	ATTATTTG	4	12 bp upstream of pyridoxine biosynthesis protein gene	430306
PittII	8	ATTATTTG	6	12 bp upstream of pyridoxine biosynthesis protein gene, 77 bp upstream of cytidylate kinase gene	1077736
22.1.21	8	ATTATTTG	4	12 bp upstream of pyridoxine biosynthesis protein gene, 77 bp upstream of cytidylate kinase gene	1370808
10810	8	ATTATTTG	6	12 bp upstream of pyridoxine biosynthesis protein gene, 77 bp upstream of cytidylate kinase gene	1862249
PittGG	9	CTTGTTTTT	6	8 bp within/13 bp upstream of low similarity to O-antigen polymerases encoding gene	1453483

Analysis of the twelve additional *Hi* genomes identified 43 non-tetranucleotide SSRs that could potentially mediate phase variation.

a The number of tandem repeat units that comprise the SSR.

b The base pair at which the 5′ base of the SSR is located.

c Location of the SSR relative to, and description of the function of, the ORF whose expression it is proposed to modulate. FS: ORF associated with SSR has a frameshift mutation, bp: base pair.
