# Supplementary material for: Simple sequence repeats in Haemophilus influenzae
Source: Infect Genet Evol. 2009 Mar;9(2):216–28. doi: 10.1016/j.meegid.2008.11.006 (PMC2651432; doi:10.1016/j.meegid.2008.11.006)
Supplement: Supplementary file 1 [file mmc1.pdf]

	gene/locus ID	Associated gene		approx dist	rpt unit	Strain: Rd	Strain: 2866	Strain: 2846	Strain: 86-026	class
	**NON-CODING REGION ASSSOCIATED REPEATS**									
mononucleotides	2866 (404270)	*tatA*	HI_0187(pseudo in Rd)	83	A	$(A)_6$	$(A)_9$	$(A)_6$	$(A)_6$	2
	Rd (134961)	*hypo/hypo*	near 3' end of HI_0119/HI_0120	n/a	T	$(T)_9$	$(T)_4C(T)_3G$	$(T)_4C(T)_3G$	$(T)_4C(T)_3G$	2
	2866 (450059)	*N/A*	dwnstrm of omp2	n/a	A	$(A)_{10}$	$(A)_9$	$(A)_7$	$(A)_7$	2
	Rd (581844)	*hypo/hypo*	HI_0561HI_0562	206/98	T	$(A)_9$	$(A)_8$	$(A)_8$	$(A)_8$	2
	86-028NP (232482)	*acpP*	NTHI0243	230	C	$(C)_5$	$(C)_5$	$(C)_5$	$(C)_{11}$	2
	Rd (723771)	*ilvY/ilvC*	HI_0681/HI_0682	528/139	A	$(A)_9$	$(A)C(A)_7$	$(A)C(A)_7$	$(A)C(A)_7$	2
	Rd (881589)	*transglycosylase*	HI_0831	58	T	$(T)_9$	$(T)_9$	$(T)_2G(T)_6$	$(T)_9$	2
	86-028NP (1145509)	*hypo*	NTHI1201	122	C	no match	$(C)_2A(C)A(C)_3$	$(C)_2A(C)A(C)_3$	$(C)_9$	2
	Rd (1062153)	*tbp2/hypo*	HI_0995/HI_0996	44/43	T	$(T)_{10}$	$(T)_2C(T)_7$	$(T)_{10}$	$(T)_2C(T)_7$	2
	86-028NP (1518695)	*hypo*	NTHI1636	50	T	no match	*see 66-8	$(T)_{10}$	$(T)_{10}$	1
	Rd (1408920)	*dacB/greA*	HI_1330/HI_1331	22/132	T	$(T)_9$	$(T)_9$	$(T)_9$	$(T)_9$	1
	2866 (982298)	*fnr*	HI_1425	17	A	$(A)_7$	$(A)_9$	$(A)_7$	$(A)_7$	2
	2866 (1173753)	*hifA/B*	r2866v6.1191/r2866v6.1190c	95/123	T	no match	$(T)_9$	no match	no match	1*
	2866 (1343902)	*ABC trans*	HI_1080	55	T	$(T)_9$	$(T)_9$	$(T)_9$	$(T)_9$	1
	Rd (229573)	*prlC (oligopeptidaseA)*	HI_0213/HI_0214	90/24	T	$(T)_7$	$(T)_7$	$(T)_9$	$(T)_7$	2
	Rd (153937)	*ompP2*	HI_0139	100	A	$(A)_8T$	$(A)_8T$	$(A)_9$	$(A)_9$	2
	2846 (1225346)	*hypo*	HI_1176	27	A	$(A)_6C(A)_2$	$(A)_8$	$(A)_9$	$(A)_9$	2[(2)]
	86-028NP (1706204)	*hypo*	NTHI1853	10	A	no match	no match	no match	$(A)_{10}$	1*
	2846 (1561562)	*trmA*	HI_0848	101	A	$(A)_4C(A)_5$	$(A)_4C(A)_5$	$(A)_9$	$(A)_4C(A)_5$	2
	Rd (1415504)	*hypo*	HI_1339	185	T	$(T)_9$	$(T)_7$	$(T)_7$	$(T)_7$	2
	Rd (15406098)	*hypo*	HI_1462.1	164	A	$(A)_9$	$(A)_7$	$(A)_7$	$(A)_7$	2
	Rd (1677208)	*tyrS*	HI_1610	62	A	$(A)_9$	$(A)_8$	$(A)_8$	$(A)_8$	2
	2866(723452)	*hia*	HI_1732 Hia pseudogene	110	T	no match	$(T)_{34}$	no match	no match	1*
di-	2866 (923693)	*hypo*	2866v6.923	47	A	no match	$(A)_9$	no match	no match	1*
	2866 (82131)	*potD2/ordL*	HI_0498/HI_0499	48/27	GC	$(GC)_5$	$(GC)_5$	$(GC)_4AC$	$(GC)_3AC(GC)$	2
	86-028NP (167686)	*chypo/htpG*	HI_0104/HI_0105	54/147	TA	$(TA)TG(TA)_3$	$(TA)TG(TA)_3$	$(TA)TG(TA)_3$	$(TA)_5$	2
tri-	86-028NP (1145531)	*hypo*	NTHI1201	144	TC	$(TC)_5$	$(TC)_5$	$(TC)TT(TC)_3$	$(TC)_5$	2
	2866 (312999)	*hxuC*	HI_0262	45	ATA	$(ATA)_9$	$(ATA)_9$	degenerate	degenerate (24 bps not 27)	2
	2866 (1852642)	*haemoglobin binding protein*	HI_1565	6	ATT	$(ATT)_4$	$(ATT)AGT(ATT)_2$	$(ATT)_4$	$(ATT)_4$	2
penta-	86-028NP (804379)	*n/a*	rRNA operon	181	AAT	$(AAT)_3$	$(AAT)_5$	$(AAT)_3$	$(AAT)_4$	2
	Rd (1369478)	*hsdM/rbfA*	HI_1287***		TCGTC	$(TCGTC)_4$	$(TCGTC)_2$	$(TCGTC)_4$	TCGTC	2
	86-028NP (1596527)	*hypo*	NTHI1744	71	TGGCT	no match	no match	no match	$(TGGCT)_3$	1*
hexa-	Rd(995716)	*hypo*	HI_0870.1	261	TTATT	$(TTATT)_3$	region absent	region absent	region absent	1*
	Rd (549556)	*pgk*	HI_0525	19	TTAAAA	$(TTAAAA)_3$	$(TTAAAA)_2TTGAAA$	$(TTAAAA)_2TTGAAA$	$(TTAAAA)_2$	2
hepta-	86-028NP (624407)	*rpoD/aspA*	Rpt 3' end of both genes	92/199	CTTTTA	$(CTTTTA)_2$	$(CTTTTA)_2$	$(CTTTTA)_2$	$(CTTTTA)_3$	2
	2866 (366328)	*n/a*	dwnstrm of HsdR2 Type I RE	n/a	TAATTCA	$(TAATTCA)_2$	$(TAATTCA)_5$	$(TAATTCA)_8$	TAATTCA	2
	2846 (737978)	*hmw2A*	2846.735c	109	TGAAAGA	no match	no match	$(TGAAAGA)_{16}$	$(TGAAAGA)_{23}$	2
	2846 (1122093)	*hmw1A*	2846.1135	110	TGAAAGA	no match	no match	$(ATCTTTC)_{17}$	$(ATCTTTC)_{17}$	1
octa-	86-028NP (1636265)	*hypo*	NTHI1794	13	AACAACC	AACAACC	$(AACAACC)_6$	AACAACC	$(AACAACC)_6$	2
	2866(816134)	*chypo/cmk*	HI_1646/HI_1647	12/80(400)	ATTATTTG	$(ATTATTTG)_2$	$(ATTATTTG)_6$	$(ATTATTTG)_4$	(ATTATTTG)GTTATTTG	2[(2)]
nona-	86-028NP (141547)	*hypo/hypo*	NTHI0160/NTHI0161	1081/85	ATTAAGAC	no match	no match	no match	$(ATTAAGAC)_3$	1*
	86-028NP (1370900)	*HMW2C*	NTHI1448	92	GTTTTCTTA	no match	no match	no match	$(GTTTTCTTA)_{19}$	1*
	Rd (292209)	*heme-hemopexin*	HI_0262	43	ATTATTATT	$(ATTATTATT)_3$	$(ATTATTATT)_3$	TAATTATCATTATT	TTATTATTATCATC	2
	Rd (1789537)	*hypo*	HI_1717	86	CGCCTTGTT	$(CGCCTTGTT)_4$	region absent	region absent	region absent	1*
	**CODING REGION ASSSOCIATED REPEATS**									
mononucleotides	HI_0285	*menF*			A	$(A)_5C(A)_4$	$(A)_{10}$	$(A)_5C(A)_4$	$(A)_5C(A)_4$	2
	HI_0021	*citG*			A	$(A)_9$	$(A)_9$	$(A)_9$	$(A)_9$	1
	HI_0513	*hindIIM*			A	$(A)_9$	region absent	region absent	region absent	1*
	HI_0648	*mdaB*			T	$(T)_9$	$(T)_7$	$(T)_6$	$(T)_6$	2[(1)]
	NTHI0546	*smb*			A	$(A)_5G(A)_3$	$(A)_5G(A)_3$	$(A)_5G(A)_3$	$(A)_9$	2
	NTHI0694	*hypo*			G	region absent	region absent	region absent	$(G)_{12}$	1*
	HI_1378	*phoR*			A	$C(A)_8$	$(A)_9$	$(A)_9$	$(A)_9$	2
	HI_1588	*purU*			A	$(A)_9$	$(A)_9$	$(A)_9$	$(A)_9$	1
	HI_1095	*dsbE*			A	$(A)_9$	$(A)_9$	$(A)_9$	$(A)_9$	1
	HI_1060	*lpxB*			A	$(A)_4G(A)_4$	$(A)_9$	$(A)_2C(A)G(A)_4$	$(A)_2C(A)G(A)_4$	2
	HI_0708	*selA*			A	$(A)_9$	$(A)_7CT$	$(A)_9$	$(A)_9$	2
	HI_1559	*hemK*			A	$(A)_9$	$(A)_7C(A)$	$(A)_7C(A)$	$(A)_7C(A)$	2
	Integrated plasmid				A	region absent	$(A)_9$	region absent	no match	1*
		hypo			A	region absent	$(A)_9$	region absent	no match	1*
dinucleotides	HI_0454	*ycfH*			GC	$(GC)_2AC(GC)AC$	$(GC)_5$	$(GC)AC(GC)_3$	$(GC)ACGT(GC)_2$	2
	HI_0008	*fdxI*			AT	$(AT)_5$	$(AT)_5$	$(AT)_5$	$(AT)_5$	1
	n/a	*lppL*			GA	$(GA)_2AA(GA)AA$	$(GA)_5$	$(GA)_5$	$(GA)_5$	2
	HI_0739	*dnaE*			CA	$(CA)_5$	$(CA)CG(CA)_3$	$(CA)_5$	$(CA)_5$	2
	HI_0735	*lpxH*			AC	$(AC)_5$	$(CA)_3TACG$	$(AC)_5$	$(AC)_5$	2

trinucleotides	HI_0579	*fusA*			ACT	(ACT)$_4$	(ACT)$_4$	(ACT)$_4$	ACC(ACT)$_3$	2
	HI_0392	*putative acetylase*			ATT	(ATT)$_4$	(ATT)ACT(ATT)$_2$	(ATT)ACT(ATT)$_2$	(ATT)ACT(ATT)$_2$	2
	HI_0229	*pnp*			CAA	(CAA)$_4$	(CAA)$_4$	(CAA)$_4$	(CAA)$_4$	1
	HI_0139	*omp2*			CAA	no match	(CAA)$_4$	no match	no match	1*
	NTHI0694	*hypo*			GGG	no match	no match	no match	(GGG)$_4$	1*
	HI_0738.1	*ilvA*			TGG	(TGG)$_4$	(TGG)CGG(TGG)CGG	CGGCGG(TGG)$_2$	CGG(TGG)$_3$	2
	HI_1070	*hrpA*			CGT	(CGT)$_4$	(CGT)$_3$	(CGT)$_3$	(CGT)$_3$	2
	HI_1696	*lsgE*			GAT	(GAT)$_4$	(GAT)$_4$	(GAT)$_4$	(GAT)$_4$	1
	HI_1139	*murC*			TGT	(TGT)$_4$	(TGT)$_4$	(TGT)$_4$	(TGT)$_4$	1
	HI_0649	*rep*			CAA	(CAA)$_4$	(CAA)$_4$	(CAA)$_4$	(CAA)$_4$	1
	HI_0221	*guaB*			GCA	GCA(GCT)$_2$GCA	GCA(GCT)$_2$GCA	(GCA)$_4$	(GCA)$_4$	2
	HI_1663	*conserved hypothetical*			TAT	(TAT)$_4$	(TAT)$_3$TCT	(TAT)$_4$	(TAT)$_3$TCT	2
	HI_1645	*fbp*			GAA	(GAA)$_4$	(GAA)$_2$GAG(GAA)	(GAA)$_4$	(GAA)$_2$GAG(GAA)	2
	HI_0661	*haemoglobin binding protein*			ATT	(ATT)$_4$	(ATT)$_4$	(ATT)$_4$	(ATT)AGT(ATT)$_2$	2
tetranucleotides	HI_1056	*hypothetical*			ATG	(ATG)$_2$ACG	ACG(ATG)ACG	(ATG)$_4$	ACG(ATG)ACG	2
	HI_0258	*lgtC*			GACA	22	26	20	10	3
	HI_0352	*lic3A*			CAAT	33	20	26	19	3
	HI_0550	*lic2A*			CAAT	23	5	25	14	3
	HI_0635	*hgpC*			CCAA	37/21	28	25	20	3
	HI_0661	*hgpB*			CCAA	20	27	39/28	12	3
	HI_0687	*fusobacteria drug/metabolite exporter*			TTTA	6				1*
	HI_1058	*mod*				(TGAC)$_{32}$	(AGCC)$_{16}$			3*
	Hi_1386 (upstream)	*putative glycosyltransferase*			CCAA	16	8	12	13	3
		yadA			GCAA	25	13	24	14	3
	HI_1537	*licA*			CAAT	17	36	7	15	3
	HI_1565	*hemoglobin binding*			CCAA	19				1*
	r2846v6.916	*pgt1*			GACA			16	14	3
	r2846v6.1528c	*lpt3 region*			AGTC			14		1*
	r2846v6.1683	*lex2A*			GCAA		24	17	14	3
	r2866v6.124c	*lav*			GCAA		20		30	3
	r2846v6.202	*oafA*			GCAA		9	14	8	3
		hgpD			CCAA				17	1*
pentanucleotides	NTHI1034	*lic3B*			CAAT				18	1*
	HI_1700	*lsgA*			TTATT	(TTATT)$_3$	(TTATT)$_3$	(TTATT)$_3$	(TTATT)$_3$	1
	HI_0936	*nrfE*			TTTAT	(TTTAT)$_3$	(TTTAT)$_3$	(TTTAT)CTTAT(TTTAT)	(TTTAT)$_3$	2
	HI_1134	*murF*			TTACC	(TTACC)$_3$	TTAGT(TTACC)TTATC	TTAGT(TTACC)TTATC	TTAGT(TTACC)TTATC	2
	HI_0867	*hmg*			TCAGC	(TCAGC)$_2$	(TCAGC)$_3$	(TCAGC)$_2$	region absent	3
hexanucleotides	2846.1264c				TCATT	TCATT	TCATT	(TCATT)$_7$	TCATT	2(2)
	HI_0264	*hxuA*			GAAAGA	no match	(GAAAGA)$_5$	no match	no match	1*
	HI_0176	*rluD*			CGTTTA	(CGTTTA)$_3$	(CGTTTA)$_3$	(CGTTTA)$_3$	(CGTTTA)$_3$	1
	HI_1232	*aceF*			GCTTCA	GCATCAGCTCCA(GCTTCA)	(GCTTCA)$_3$	GCATCAGATTCAGCATCA	GCATCA(GCTTCA)ACATCA	2
	HI_1264	*gyrA*			AGATGA	(AGATGA)$_2$	(AGATGA)$_2$	(AGATGA)$_3$	(AGATGA)AGAAGA	2
	HI_0995	*tbp*			AAAAAG	no match	AAA(AAAAAG)$_2$	(AAAAAG)$_3$	no match	2(2)
	HI_0251	*tonB*			AGCCAG	(AGCCAG)$_4$	(AGCCAG)AGCCTG(AGCCAG)	(AGCCAG)AGCCTG(AGCCAG)	(GACCGA)GTCCGA$_2$	2
	HI_0264	*hxuA*			AATGGC	(AATGGC)$_3$	AAGGGC(AATGGC)AATAGC	(AATGGC)AATACC$_2$	(AATGGC)$_3$	2
hepta-	HI_0917	*protective surface antigen D15*			ATGGTA	(ATGGTA)$_3$	ATGATA(ATGGTA)ATAGTTCTGGTA	(ATGGTA)GCGGTA(ATGGTA)ATGGTG	(ATGGTA)$_3$	2
nona-	HI_1042	*metH*			ACGGCTT	ACCGCTT(ACGGCTT)	ACCGCTT(ACGGCTT)	(ACGGCTT)$_3$	region absent	2
	2846.733	*pseudo gene*			TTGCTTGGT	region absent	region absent	(TTGCTTGGT)$_{12}$	TTGCTTGGT	2(2)
	2866 (1558354)	*hmg locus*			CTTGTTTTT	no match	(CTTGTTTTT)$_3$	CTTCGCTTT(CTTGTTTTT)	region absent	2(2)
								**class 1**	*did not vary in any*	
								**class 1***	*only data on one strain*	
								**class 2**	*change in repeat unlikely to lead to PV phenotype*	
								**2(2)**	*variability cannot say for sure non-PV phenotype*	
								**class 3**	*change consistent with PV phenotype*	
